# Proceedings from the CIH-LMU 2021 Symposium: “Global Health Perspectives: Climate Change & Migration”

**DOI:** 10.1186/s12919-021-00225-4

**Published:** 2021-10-12

**Authors:** Wandini Lutchmun, Aikins Ablorde, Han-Wen Chang, Guenter Froeschl, Equlinet Misganaw, Bhim Prasad Sapkota, Sarah Scholze, Josephine Singo, Lisa Hoffaeller

**Affiliations:** 1 Teaching & Training Unit, Division of Infectious Diseases and Tropical Medicine, University Hospital, LMU, Munich, Germany; 2 Center for International Health, Ludwig-Maximilians-Universität, Munich, Germany

**Keywords:** Climate Change, Climate migration, Global Health, Climate resilience, Climate justice

## Abstract

Climate change shapes human migration through the interaction of environmental changes with political, social, economic, and demographic drivers of mobility. Low-and middle-income countries bear the brunt of the health impacts of climate change and migration, despite their overall low contribution to greenhouse gas emissions. The CIH^LMU^ Symposium 2021 aimed to explore the complex interconnections between climate change, migration and health from diverse global perspectives. A number of themes, such as the relationship between climate and trade, the role of technology, and the issue of responsibility were tackled. The speakers also highlighted the need for climate resilient health-systems, gender mainstreaming in climate strategies, collaboration between the Global North and South and urgently defining the ‘climate refugee’. It is crucial that the narrative around climate change moves from an environmental framing to encompass human health and migration within climate discussions and strategies.

## Introduction

By 2050, 200 million people globally are projected to be displaced as a result of climate change [1]. Slow onset climate processes, such as sea-level rise, food and water insecurity and desertification; and fast climate events, such as floods and hurricanes [2]; impose direct and indirect risks to human health. These risks interact with pre-existing low resilience and adaptive capacity, lack of development and low preparedness, to drive human migration. The relationship between climate change, migration and health is heterogenous [3] and mediated by context-dependent political, social, economic, and demographic drivers of mobility [4]. While migration is often an adaptive solution to climate change, it can also be maladaptive, and negatively impact human health [5]. The climate-migration-health nexus was explored from diverse perspectives at the Center for International Health of the Ludwig Maximilians Universität (CIH^LMU^) 2021 Symposium “Global Health Perspectives: Climate Change and Migration” on March 12th, 2021.

## Methods

### Institutional framework

The symposium “Global Health Perspectives: Climate Change & Migration” was conceived as an event in a series of symposia in the field of International Health conducted annually since 2012 at CIH^LMU^ in Munich, Germany. The series serves both as scientific events tackling current topics in International Health, and as part of the study curriculum for students of the PhD Program Medical Research-International Health and the MSc Program in International Health. The majority of the students originate from low- and middle-income countries (LMICs) and conduct research projects targeting health challenges in their home countries. For the successful conception, organization and delivery of the symposium, students gain European Credit Transfer System (ECTS) points.

### Organizational framework

The organizational group consisted of four PhD candidates and two MSc students. The group was facilitated by the PhD and MSc coordinators and administrative staff of the CIH^LMU^ and received coaching by an expert in Project Management and Intercultural Communication throughout the preparatory process.

### Content selection

Health in the context of climate change and migration was considered a topic of high relevance and importance by the students at the time of topic selection. A pool of prospective speakers was prepared based on professional expertise and geographical background, and the speakers were invited to suggest a contribution based on the thematic objectives of the event.

### Event delivery

Until 2020 the symposium was conducted in person in the auditorium of the LMU in Munich. Due to the COVID-19 pandemic, this event was held online on March 12, 2021, using the online communication tool ‘Zoom Meetings’. Registration was free of charge and open from the 4th of February to the 11th of March 2021. During the event, each speaker delivered a 30-min presentation, followed by a 15-min ‘Question & Answer’ session where participants’ questions, delivered via the chat function, were answered. This was followed by a panel discussion moderated by a guest speaker. MSc and PhD students of the organising committee also shared their research projects via 5-min presentations. Symposium attendees holding a medical license were eligible for 6 Continuous Medical Education (CME) credit points.

## Summary of presentations

### “Climate change on the move”

#### Peter van den hazel, MD, PhD, MPH

Environmental health consultant in Arnhem, Netherlands, President of the Health and Environmental Alliance (HEAL), Brussels, Belgium, and Chair of the International Network on Children’s Health and Safety (INCHES), Ellecom, Netherlands.

##### Overview

The health impacts of climate change are unequally distributed at three levels. At the macro, or global level, LMICs and disadvantaged minority settings bear the highest burden of disease, predominantly from extreme weather events and air pollution. At the meso, or local level, residents of ecologically fragile areas and rapidly growing urban cities suffer higher health risks, often as a result of air and water pollution. At the micro level, health risks are determined by individual factors and personal environment, one particularly vulnerable population being children.

Globally, three leading causes of under 5 mortality are diarrheal illness, respiratory tract infections and malaria [6], 98% of which occur in low-income countries [7], and all of which are heavily impacted by a changing climate. Changes in temperature and rainfall patterns and the resulting changes in vector ecology; contaminated drinking water as a result of extreme weather events; and air pollution, all contribute to the increased rates of the aforementioned diseases, exacerbated by food and water scarcity and resulting malnutrition. Children are not only physiologically vulnerable to the effects of climate change, but their vulnerability is exacerbated by their reliance on adults for survival and development [8]. Up to 175 million children are affected by climate disasters every year, and children make up a third of refugees, internally displaced people (IDPs) and asylum-seekers [7]. Illness and climate-related displacement at a young age can have devastating impacts on a child’s education, development, safety and psychological well-being, the burden of which they may carry for the rest of their lives.

##### Discussion

-Research on the mental health consequences of climate change and climate migration, especially in children, is lacking severely, possibly due to the complexity of this field.

-Small island states (SISs) are particularly vulnerable to the impacts of rising sea levels and the increase in intensity and frequency of extreme weather events. The Paris agreement recognises these vulnerabilities and has set out plans for a global fund for developing countries and SISs to plan and implement their climate adaptation and mitigation strategies [9].

### “Climate change, migration and health: an African perspective”

#### Chukwumerije Okereke, PhD

Director of the Centre for Climate Change and Development at the Alex Ekwueme Federal University, Ndufu-Alike, Nigeria and visiting lecturer at the University of Reading and the Oxford University Centre for the Environment (OUCE), United Kingdom.

##### Overview

A moral approach to climate change identifies three main asymmetries. An asymmetry in contribution results from disproportionate Greenhouse Gas (GHG) emissions across countries. For example, in 2016, the average US citizen emitted about 16 tons of carbon/day while the average Congolese emitted less than 0.1 [10]. The asymmetry in the power to decide, is often exemplified by the underrepresentation of some countries during climate negotiations; for example, at United Nations Framework Convention on Climate Change (UNFCCC) Conference of Parties (COP) in 2007, Germany was represented by 101 delegates while Ethiopia’s interests were covered by only 2 delegates, despite similar populations. An asymmetry in impact is observed in the disproportionate impacts of climate change across countries, the highest impacts often felt by those contributing the least to GHG emissions.

Africa contributes less than 4% to global GHG emissions [11], yet is one of the most vulnerable continents to the effects of climate change. Coastal nations of West and Central Africa, home to many rapidly growing economic hubs, have low-lying lagoons threatened by sea level rise and therefore erosion, inundation and flooding [12]. Flooding events in Nigeria have already led to large displacements, such as the Niger Delta Flood Disaster in 2012, which forced 2.1 million people to flee their homes [13]. Furthermore, the vulnerability of food production systems in Africa to climate change will result in a decline in important economic crops such as Banana and Plantain in West Africa, while Southern Africa is estimated to suffer crop yield losses of 18% by 2050 [12]. Desertification, as in the case of the drastic shrinkage of the Lake Chad basin, has led to the southern migration of cattle herders and farmers in search of greener pastures, causing issues of conflict. Pre-existing inadequate health and sanitation infrastructure, water systems and access to health services also exacerbate the health risks of climate change. The growing population, rapid rates of urbanisation, especially in coastal areas, coupled with a heavy reliance on agriculture, poor infrastructure, and poor governance all act to reduce Africa’s adaptive capacity to a changing climate [12].

##### Discussion

-China is currently the global leader in total GHG emissions, followed by the United States [14]. It is important to recognise the increasing contribution to GHG emissions from LMICs in the past decades and encourage collaboration between LMICs and HICs in planning and implementing mitigation and adaptation strategies and sustainable development.

-Technology has long been contested as either an important contributor to climate change or part of the climate solution. However, technological innovations in the past 10 years have been the most important developments in tackling climate change.

### Developing climate resilient health systems in developing countries

#### Meghnath Dhimal, PhD

Chief Research Officer at the Nepal Health Research Council and Coordinator of the Young Scientists Forum of Nepal.

##### Overview

Nepal is highly vulnerable to the effects of climate change, due to its complex topography, climate variability, high risk of climate disasters and low socioeconomic status [15]. The NAP (National Adaptation Plan) is a government-led process, established in Nepal after the COP 2010 in Cancun, to facilitate the planning and implementation of sustainable medium- and long-term adaptation programmes [16]. In 2016, Nepal ratified the Paris Climate agreement and its Nationally Determined Contribution (NDC) and implemented the H-NAP, the health component of the NAP, guided by the “WHO operational framework for building climate resilient health systems” [15]. Furthermore, by signing the Male Declaration at the 70th WHO Regional Committee for South-East Asia in 2017, along with the other 10 member states of the WHO South-East Asia Region, Nepal made a regional commitment to building a climate change resilient health system. Nepal is involved in regional projects such as “Delivering climate-resilient water and sanitation in Africa and Asia”, and “Building resilience of health systems in Asian LDCs (least-developed countries) to climate change”. National capacity building is key for adaptation and developing resilience to climate change through implementation of climate resilient health systems and increasing capacity for disaster preparedness and response.

##### Discussion

-The Green Climate Fund (GCF) has made commitments to fund climate adaptation projects in Nepal, with some additional financial support from the European Union and the United Kingdom. However, the overall support from developed nations remains low.

-The effects of climate change are not gender neutral and perspectives from the sphere of social sciences are often lacking in climate discussions and solutions, especially in LMICs. Nepal has recently established a thematic group for gender mainstreaming in climate change adaptation and mitigation strategies.

- Consumption behaviours at the societal but also individual level need to be challenged and shifted to more sustainable practices. A standard cup of coffee, for example, has a water footprint of 130 l [17], while the production of just one cotton shirt requires approximately 3000 l of water [18].

### Trends, magnitude and distribution of the effects of climate change & migration: a Canadian perspective

#### Shelby Yamamoto, PhD

Assistant Professor at the School of Public Health, University of Alberta, Canada and Principal Investigator of the projects “Climate change, older adults and immigrants: exploring community vulnerability and resilience” and “Climate change surveillance for chronic health effects in vulnerable populations”.

##### Overview

Canada is warming at twice the global rate [19], resulting in a wide range of health effects and changing patterns of migration. One particularly affected group are the Indigenous peoples of Canada that include Inuit, Métis, First Nations, and all First Peoples [20]. Indigenous communities face unique challenges as their livelihoods depend on their spiritual, cultural and environmental ties to the land [21]. This is also exacerbated by marginalization, living in remote, inaccessible areas, often with poor surrounding infrastructure, and an ageing population, which can be among the drivers of relocation to other communities or urban areas [20]. The warming climate has also affected the migratory patterns of animal species and led to changes in harvesting patterns, fueling food insecurity among the Indigenous populations who may rely on subsistence hunting and traditional foods. Another important group to consider in terms of climate-change related health impacts in Canada are immigrants, a significant proportion of whom originate from LMICs. Many contextual factors, such as the nature of the migration process, the capacity of the receiving community, and other push-pull factors, influence the health outcomes of immigrant populations [22]. Research in this area is heterogenous and lacking. While demographic studies have highlighted “the healthy immigrant-effect”, due to the positive selection of healthier people to migrate, this converges to host country levels over time [22]. Research has shown that immigrants, especially those migrating from rural to urban areas, have higher rates of chronic illness [22]. Immigrants, a heterogenous group, often face numerous challenges such as cultural and language barriers, discrimination, socioeconomic differences, lifestyle changes, changes in their living arrangements, occupational exposures as well as barriers to accessing healthcare, which can result in poor health. While migration can pose significant health risks, it is an important adaptation response to the effects of a changing climate. The health needs of migrant and Indigenous populations should be at the forefront of climate adaptation strategies in Canada.

##### Discussion

-History and background are important considerations when developing climate change interventions and strategies that promote climate change adaptation and resilience.

-Indigenous-led research and partnerships are key in addressing climate change. This helps set priorities and needs as determined by communities and capture indigenous knowledge.

-More focus needs to be put on potential mental health challenges faced by the immigrant populations in Canada and addressing stigma as a barrier to seeking help.

## Panel discussion

### Moderator: Martin Herrmann, MD

Founder and spokesperson of the German Alliance for Climate Change and Health (*Deutsche Allianz Klimawandel und Gesundheit*).

### Panelists: Peter van den hazel, Chukwumerije Okereke, Shelby Yamamoto, Meghnath Dhimal

-Migration is a global phenomenon and while estimates show that the majority of global migration occurs between or within LMICs, the dialogue, and research, tends to focus almost entirely on migration from LMICs to high income countries (HICs) [23].

-At the policy level, the Intergovernmental Panel on Climate Change (IPCC) will be crucial in pushing the migration and health agenda. It is important to integrate migration and health not just within adaptation regimes but also within the Loss and Damage (L&D) regime of the IPCC, however, political awareness, and interests, are often lacking.

- The “climate refugee” is currently not defined by International Law, due to its hermeneutic difficulty, and vast political implications, one being the obligation of developed countries to confer the same protection to climate refugees as to political refugees. A ‘refugee’ also crosses international borders, which does not recognise IDPs, who make up the majority of climate migrants. Furthermore, since climate change is often interwoven amongst other drivers of mobility, the decision of who is a climate refugee or not can be extremely difficult to make [2].

-Trade and climate change are closely linked. Trade affects emissions through its influence on consumption and investment patterns, relocation of production (often to LMICs) and international transport but also on transfer of technology [24]. China ranks as the highest global contributor to pollution by total emissions per annum, however as a net exporter of emissions, a large amount of China’s emissions is embedded in produced goods exported and consumed by developed countries such as the United States [14]. Trade rules need to tackle the issue of embedded carbon, without compromising the rights of developing countries towards economic development.

-While GHG emissions incurred a drop during the COVID-19 pandemic in 2020, the effects are negligible on the larger scale of climate change [25]. The economic impacts of COVID-19 in Africa will be catastrophic; projections show that millions of Nigerians will be pushed into poverty endangering Nigeria’s chance of reaching its Sustainable Development Goals (SDGs).

-The COVID-19 pandemic has also shown that governments are able to make drastic decisions in acute situations. Lessons learnt from the pandemic, such as avoiding delayed action, enhancing community engagement, addressing inequality and promoting international collaboration [26], can help guide climate mitigation strategies.

-The integration of climate change into medical education is essential in forming health professionals knowledgeable of the changing patterns of disease caused by climate change and migration.

## Conclusion

The climate-migration-health nexus is complex and heterogeneous. Children are particularly vulnerable both to the short- and long-term consequences of climate change and should be at the core of climate adaptation strategies. Furthermore, the definition of a climate refugee is critical for the protection of the vulnerable populations displaced nationally and globally as a consequence of climate change. LMICs bear the highest burden of impacts despite their overall low contribution to GHG emissions but are still lacking the technical competencies and the necessary bargaining powers to mitigate, and adapt to, the effects of changing climate. Concurrently, the growing GHG emissions from LMICs in the last few decades has highlighted the need for sustainable economic development using green solutions and energy, through collaboration with HICs.

## Data Availability

NA

## Abbreviations

CIH^LMU^: Center for International Health of the Ludwig Maximilians Universität
CME: Continuous Medical Education
COP: Conference of Parties
ECTS: European Credit Transfer System
GCF: Green Climate Fund
GHG: Greenhouse Gas
HEAL: Health and Environmental Alliance
HIC: High-income country
HNAP: Health National Adaptation Plan
IDP: Internally Displaced Person
INCHES: International Network on Children’s Health and Safety
IPCC: Intergovernmental Panel on Climate Change
L&D: Loss and Damage
LDC: least-developed country
LMIC: Low-and middle-income country
NAP: National Adaptation Plan
NCD: Nationally Determined Contribution
OUCE: Oxford University Centre for the Environment
SDG: Sustainable Development Goal
SIS: Small Island State
UNFCCC: United Nations Framework Convention on Climate Change
WHO: World Health Organization

## References

[1] Myers N (2002). Environmental refugees: a growing phenomenon of the 21st century. Philos Trans R Soc Lond Ser B Biol Sci.

[2] Brown O (2008). Migration and climate change.

[3] Schwerdtle PN, McMichael C, Mank I, Sauerborn R, Danquah I, Bowen KJ (2020). Health and migration in the context of a changing climate: a systematic literature assessment. Environ Res Lett.

[4] Black R, Adger WN, Arnell NW, Dercon S, Geddes A, Thomas D (2011). The effect of environmental change on human migration. Glob Environ Change..

[5] Schütte S, Gemenne F, Zaman M, Flahault A, Depoux A (2018). Connecting planetary health, climate change, and migration. Lancet Planet Health.

[6] Liu L, Oza S, Hogan D, Chu Y, Perin J, Zhu J, Lawn JE, Cousens S, Mathers C, Black RE (2016). Global, regional, and national causes of under-5 mortality in 2000–15: an updated systematic analysis with implications for the sustainable development goals. Lancet..

[7] Venton CC. The benefits of a child-centred approach to climate change adaptation. UNICEF and Plan International; 2011.

[8] UNICEF. Unless We Act Now: The Impact of Climate Change on Children; 2015.

[9] The HD (2015). Paris climate agreement: outcomes and their impacts on small island states. Island Stud J.

[10] World Bank, World Development Indicators. CO2 emissions (metric tons per capita). The World Bank. 2017. https://data.worldbank.org/indicator/EN.ATM.CO2E.PC.

[11] Fields S (2005). Continental divide: why Africa’s climate change burden is greater. Environ Health Perspect.

[12] Niang I, Ruppel OC, Abdrabo MA, Essel A, Lennard C, Padgham J, Urquhart P. Africa. In: Barros, V.R., Field, C.B., Dokken, D.J., Mastrandrea, M.D., Mach, K.J., Bilir, T.E., Chatterjee, M., Ebi, K.L., Estrada, Y.O., Genova, R.C., Girma, B., Kissel, E.S., Levy, A.N., MacCracken, S., Mastrandrea, P.R. and White, L.L., Eds., Climate Change 2014: Impacts, Adaptation, and Vulnerability. Part B: Regional Aspects. Contribution of Working Group II to the Fifth Assessment Report of the Intergovernmental Panel on Climate Change, Cambridge University Press, Cambridge. 2014:1199–265.

[13] Amadi L, Mac Ogonor CU (2015). Climate change, environmental security and displacement in Nigeria: experience from the Niger Delta flood disaster, 2012. Afr J Environ Sci Technol.

[14] Ritchie H, Roser M. CO_2_ and Greenhouse Gas emissions. Our World in Data. 2020. https://ourworldindata.org/co2-and-other-greenhouse-gas-emissions?country=. Accessed 2 Jun 2021.

[15] Dhimal M, Dhimal ML, Pote-Shrestha RR, Groneberg DA, Kuch U (2017). Health-sector responses to address the impacts of climate change in Nepal. WHO South East Asia J Public Health.

[16] Ebi KL, Prats EV (2015). Health in National Climate Change Adaptation Planning. Ann Glob Health.

[17] Mekonnen MM, Hoekstra AY (2011). The green, blue and grey water footprint of crops and derived crop products. Hydrol Earth Syst Sci Discuss.

[18] Chapagain AK, Hoekstra AY, Savenije HHG, Gautam R (2006). The water footprint of cotton consumption: an assessment of the impact of worldwide consumption of cotton products on the water resources in the cotton producing countries. Ecol Econ.

[19] Bush E, Lemmen DS (2019). Canada’s changing climate report.

[20] Ford JD, Berrang-Ford L, King M, Furgal C (2010). Vulnerability of Aboriginal health systems in Canada to climate change. Glob Environ Change.

[21] Ford JD, King N, Galappaththi EK, Pearce T, McDowell G, Harper SL (2020). The resilience of indigenous peoples to environmental Change. One Earth.

[22] McMichael C, Barnett J, McMichael AJ (2012). An ill wind? Climate change, migration, and health. Environ Health Perspect.

[23] Abubakar I, Aldridge RW, Devakumar D, Orcutt M, Burns R, Barreto ML, Dhavan P, Fouad FM, Groce N, Guo Y, Hargreaves S, Knipper M, Miranda JJ, Madise N, Kumar B, Mosca D, McGovern T, Rubenstein L, Sammonds P, Sawyer SM, Sheikh K, Tollman S, Spiegel P, Zimmerman C, Abubakar I, Aldridge RW, Devakumar D, Orcutt M, Burns R, Barreto ML, Dhavan P, Fouad FM, Groce N, Guo Y, Hargreaves S, Knipper M, Miranda J, Madise N, Kumar B, Mosca D, McGovern T, Rubenstein L, Sammonds P, Sawyer SM, Sheikh K, Tollman S, Spiegel P, Zimmerman C, Abbas M, Acer E, Ahmad A, Abimbola S, Blanchet K, Bocquier P, Samuels F, Byrne O, Haerizadeh S, Issa R, Collinson M, Ginsburg C, Kelman I, McAlpine A, Pocock N, Olshansky B, Ramos D, White M, Zhou S (2018). The UCL–Lancet Commission on migration and health: the health of a world on the move. Lancet..

[24] Blanco G, R Gerlagh, S Suh JB, de Coninck C, Diaz Morejon F, Mathur R, et al. Chapter 5: Drivers, Trends and Mitigation. In: Climate Change 2014: Mitigation of Climate Change. Contribution of Working Group III to the Fifth Assessment Report of the Intergovernmental Panel on Climate Change. Cambridge University Press, Cambridge, United Kingdom and New York, NY, USA.; 2014.

[25] Le Quéré C, Jackson RB, Jones MW, Smith AJP, Abernethy S, Andrew RM (2020). Temporary reduction in daily global CO 2 emissions during the COVID-19 forced confinement. Nat Clim Chang.

[26] Klenert D, Funke F, Mattauch L, O’Callaghan B. Five Lessons from COVID-19 for Advancing Climate Change Mitigation. Environ Resour Econ. 2020;76:751–78. 10.1007/s10640-020-00453-w PMC7397958 32836842

